# Heterozygote advantage and pleiotropy contribute to intraspecific color trait variability

**DOI:** 10.1111/evo.14597

**Published:** 2022-08-29

**Authors:** Chiara De Pasqual, Kaisa Suisto, Jimi Kirvesoja, Swanne Gordon, Tarmo Ketola, Johanna Mappes

**Affiliations:** ^1^ Department of Biological and Environmental Science University of Jyväskylä Jyväskylä 40014 Finland; ^2^ Organismal and Evolutionary Biology Research Program University of Helsinki Helsinki 00014 Finland; ^3^ Department of Ecology and Evolutionary Biology Cornell University Ithaca New York 14853

**Keywords:** Color locus, heterozygote advantage, intraspecific trait variation, life‐history traits, pleiotropy, wood tiger moth

## Abstract

The persistence of intrapopulation phenotypic variation typically requires some form of balancing selection because drift and directional selection eventually erode genetic variation. Heterozygote advantage remains a classic explanation for the maintenance of genetic variation in the face of selection. However, examples of heterozygote advantage, other than those associated with disease resistance, are rather uncommon. Across most of its distribution, males of the aposematic moth *Arctia plantaginis* have two hindwing phenotypes determined by a heritable one locus‐two allele polymorphism (genotypes: WW/Wy = white morph, yy = yellow morph). Using genotyped moths, we show that the presence of one or two copies of the *yellow* allele affects several life‐history traits. Reproductive output of both males and females and female mating success are negatively affected by two copies of the *yellow* allele. Females carrying one *yellow* allele (i.e., Wy) have higher fertility, hatching success, and offspring survival than either homozygote, thus leading to strong heterozygote advantage. Our results indicate strong female contribution especially at the postcopulatory stage in maintaining the color polymorphism. The interplay between heterozygote advantage, *yellow* allele pleiotropic effect, and morph‐specific predation pressure may exert balancing selection on the color locus, suggesting that color polymorphism may be maintained through complex interactions between natural and sexual selection.

The origin and maintenance of polymorphism—the co‐occurrence of more than two distinct morphs—within natural populations constitute a long‐standing conundrum in evolutionary biology (Ford 1945; Huxley 1955; White 2017). Drift alone can erode phenotypic variation from populations in a few hundred generations (Nevo et al. 1997). If traits are under selection, polymorphism is even more puzzling. Theory predicts that traits contributing to the fitness of individuals should be under strong natural and stabilizing selection and drive the more fit morph to fixation (Endler, 1988; Cardé and Baker, 1984). Still, color polymorphic populations are widespread in nature (e.g., Sinervo and Lively 1996; Pryke and Griffith, 2007; Maan and Cummings, 2008; Hegna et al., 2015). Traits (i.e., coloration) may be shaped by complex evolutionary processes through multiple and nonmutually exclusive selective pressures (Gray and McKinnon, 2007), which drive and maintain phenotypic variation and genetic diversity in nature (Fisher 1930; Ford 1945).

Coloration, for example, plays an important role in a variety of ecological and physiological processes (Endler and Mappes, 2017; Cuthill et al., 2017), from camouflage (Duarte et al., 2017), to warning coloration (Mappes et al., 2005) and sexual selection (Maan and Cummings, 2008). Thus, color polymorphism may be the result of natural selection (Gray and McKinnon 2007), sexual selection (Wellenreuther et al., 2014), their combination (Maan and Cummings, 2008), and/or pleiotropic effects (i.e., when a single locus affects two or more phenotypic traits) because color morphs are often genetically correlated with other traits (McKinnon and Pierotti 2010). Alternative color morphs often differ in features other than color (McKinnon and Pierotti 2010). For example, variable morph‐specific behavioral strategies, such as territoriality (Sinervo and Lively 1996), aggressiveness and dominance (Pryke and Griffith 2007), or alternative reproductive strategies, may exist (Sinervo and Lively 1996; Tuttle 2003).

Complex phenotypes can be controlled by simple genetic mechanisms (i.e., one or few genes). In *Drosophila melanogaster*, a gene responsible for cuticle pigmentation, *yellow*, has pleiotropic effects on other traits in males. The lack of function of the *yellow* gene disrupts body pigmentation expression, male courtship behavior, and mating success (Bastock 1956; Wilson et al., 1976; Massey et al., 2019) caused by a morphological and structural change on the leg section used by the male to grasp the female (i.e., sex comb) (Massey et al., 2019). In the case of the common wall lizard (*Podarcis muralis*), the simple genetic basis of the color polymorphism leads to pleiotropic effects in numerous traits (Andrade et al., 2019), including morphology (Sacchi et al., 2007), behavior (Abalos et al., 2016), physiology (Galeotti et al., 2010), immunology (Calsbeek et al., 2010), and reproduction (Galeotti et al., 2013).

Intraspecific color polymorphism maintenance typically requires some form of balancing selection, achieved through color morph fluctuations resulting from negative frequency‐dependent selection (FDS) (Wellenreuther et al., 2014) or independent of the relative abundance of a morph (Pryke and Griffith, 2007; Hedrick et al., 2016). Negative FDS mediated by sexual selection can maintain multiple color morphs in natural populations, for example, through alternative male reproductive strategies in the side‐blotched lizards (*Uta stansburiana*) (Sinervo and Lively 1996), through rare morph advantage in guppies (*Poecilia reticulata*) (Hughes et al., 2013), or through FD sexually antagonistic selection in blue‐tailed damselflies (*Ischnura elegans*) (Svensson and Abbott, 2005; Svensson et al., 2005). In populations with stable morph frequencies, nonrandom mating, in concert with other selective forces, can prevent the loss of color morphs through within‐morph mating (i.e., assortative mating) (Pryke and Griffith 2007) or can promote morph maintenance through disassortative mating that maintains high heterozygosity and genetic variation within a population (Hedrick et al., 2016; Maisonneuve et al., 2021).

The presence of two different alleles at a locus (i.e., heterozygosity) provides a basis for phenotypic variation within populations, for example, by expressing alternative color morphs. If heterozygote individuals have a fitness advantage over the homozygote ones, the persistence of phenotypic polymorphism and genetic variability can be aided through heterozygote advantage (Fisher 1922, 1930; Hedrick 2012). Despite decades of research, the majority of studies have focused on heterozygote advantage as a phenomenon of disease resistance, especially in humans (e.g., the sickle cell anemia, Allison 1954; AIDS, Carrington et al. 1999), in the environment (e.g., pesticide resistance, Greaves et al. 1977; infection resistance, Frelinger, 1972), or to maximize fecundity in livestock (Gemmell and Slate 2006). Recently, due to the advantages of the modern molecular biological methods, there is an increasing number of studies focusing on the role of heterozygote advantage in color polymorphic wild populations (Krüger et al., 2001; Coulson et al., 2011; Hedrick et al., 2014; Llaurens et al., 2017; Strickland et al., 2021). Heterozygote advantage is not an easy task to study in wild populations. The challenges lie in gathering life‐history traits of the different genotypes and, sometimes, the lack of knowledge of the genetic basis of the polymorphic trait.

Compelling examples of the fitness advantage of heterozygote individuals are phenotypic variability of sexually selected traits (Coulson et al., 2011; Krüger et al., 2001; Johnston et al., 2013; Hedrick et al., 2014; Maisonneuve et al., 2021), concurrently with other selective forces. In the common buzzard (*Buteo buteo*), the plumage color polymorphism is maintained through heterozygote advantage, which counterbalances maladaptive assortative mate choice due to maternal sexual imprinting (Krüger et al., 2001). The color coat of wolves in Yellowstone National Park represents another well‐known example, whose stable color polymorphism maintenance is due to heterozygote advantage (Coulson et al., 2011; Hedrick et al., 2014) coupled with weak selection (Hedrick et al., 2014) and a strong contribution of disassortative mating (Hedrick et al., 2016). Complex polymorphisms can thus be maintained by the interplay of multiple selective pressures, of which heterozygote advantage is one vastly understudied mechanism, and which altogether may determine phenotype‐specific advantages culminating in the coexistence of multiple phenotypes.

The wood tiger moth (*Arctia plantaginis*) represents a compelling study species to investigate how different selective pressures can act on a single color locus and maintain within‐population trait variation. In this system, male hindwing coloration is determined by a simple genetic basis (Suomalainen 1938; Nokelainen et al., 2022b; Brien et al., 2022): a one locus‐two allele polymorphism (dominant W allele and recessive y allele), which translates into white (genotype: WW, Wy) and yellow (genotype: yy) males. Because this is an aposematic moth species, the color trait is not only used for intraspecific communication (i.e., sexual selection) but also to advertise their unpalatability to predators (i.e., interspecific communication). Previous studies have indeed shown that multiple selective pressures act on the male coloration. The two male morphs are differently protected against predators (Nokelainen et al., 2014; Rojas et al., 2017; Winters et al., 2021), with yellow males generally having higher survival (Nokelainen et al., 2012; Rojas et al., 2017). In addition, male morph mating advantage is dependent on the morph frequency (Gordon et al., 2015) and males that origin from “mixed‐morph lines” have higher mating success compared to the moths that originated from more monomorphic lines (Gordon et al., 2018), which suggests that heterozygote advantage may also contribute to the color polymorphism in this species. Here, we test the hypothesis that heterozygote advantage is contributing to male hindwing color polymorphism in the wood tiger moth. By using genotyped lines of moths reared in a greenhouse and life‐history traits collected across 19 generations (i.e., 7 years), we subjected the three color genotypes (WW, Wy, and yy) to multiple tests. We test whether heterozygote individuals have (1) higher mating success, either through higher probability of copulating (copulation observations) or lower probability of unsuccessful matings; (2) higher reproductive output by testing fecundity, fertility, and hatching success; and (3) higher longevity by testing the adults’ life span.

## Material and Methods

### STUDY SPECIES

**Figure 1:**
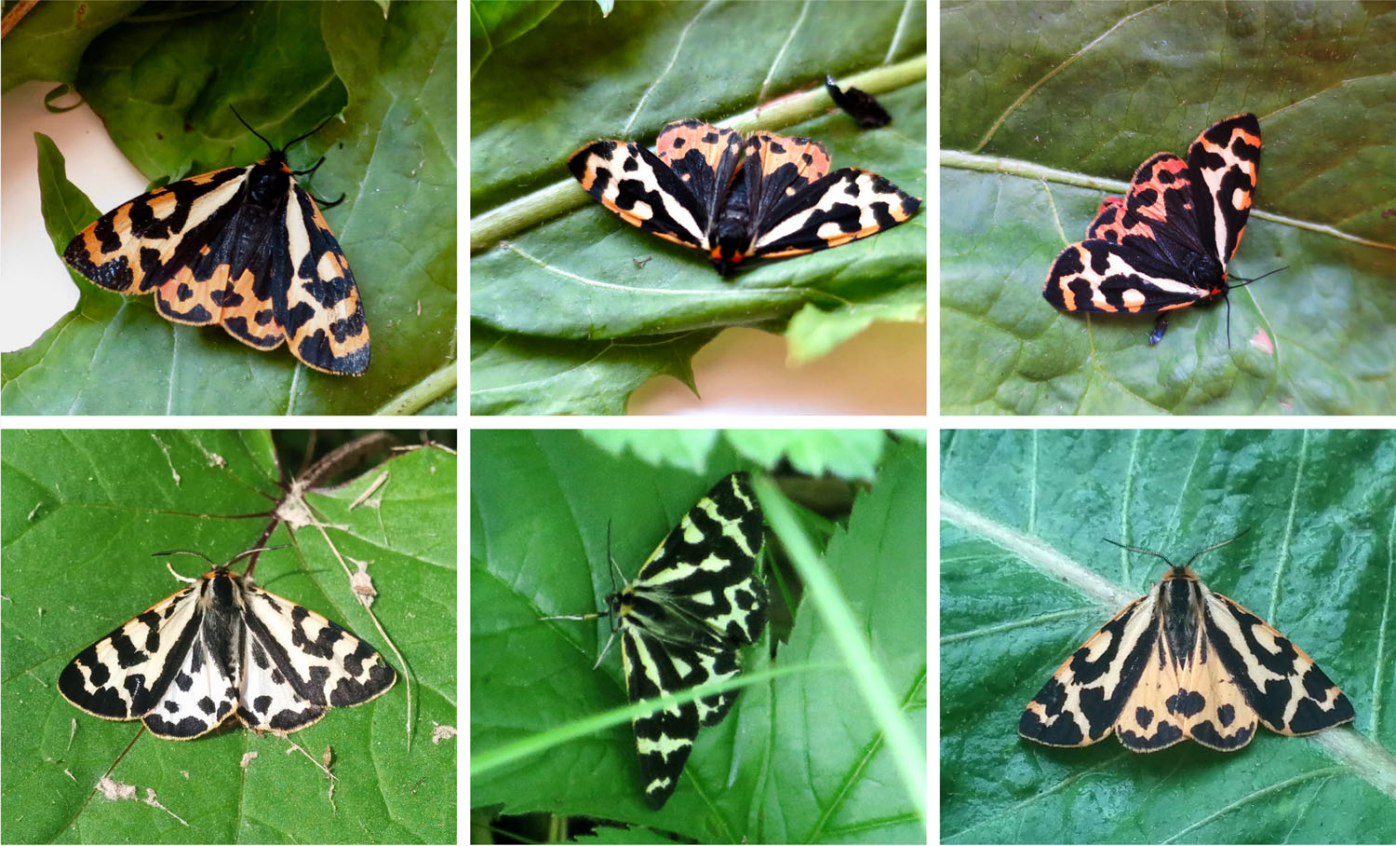
Examples of wood tiger moth hindwing coloration in Finland. Female's (top row) hindwing coloration varies from light to dark red, whereas male's (bottom row) coloration is either white (left and middle photo) or yellow (right photo). White males can either be WW or Wy for the dominant W color allele, whereas yellow males are homozygote for the y recessive allele. Photos: Chiara De Pasqual.

The wood tiger moth (*Arctia plantaginis*) (formerly *Parasemia plantaginis*; Rönkä et al., 2016) is a polymorphic and aposematic moth species. The male hindwing coloration is determined by a simple genetic mechanism where a one locus‐two allele (W and y allele) polymorphism translates into white (WW or Wy genotype) or yellow (yy genotype) male morphs (Suomalainen 1938; Nokelainen et al., 2022b) (Fig. 1). Females do not phenotypically express the male color alleles as their hindwing coloration varies continuously from yellow to red but pass the color alleles to their offspring (Nokelainen et al., 2022b) (Fig. 1). The wood tiger moth is a capital breeder; it does not feed at the adult stage, making the larval diet very important for both their development and the adult stage (e.g., sperm quality, egg numbers) (Tammaru and Haukioja 1996). Adults only live for 1 or 2 weeks after their emergence and spend their adulthood looking for suitable mates. Females lay on average 250 eggs within a few days from the copulation event. Larvae hatch after about 7 days (Chargé et al., 2016), and start feeding on a variety of weedy plants (e.g., *Plantago* sp., *Taraxacum* sp., and *Rumex* sp.).

In Finland, the wood tiger moth has one generation per year and the flight season happens between mid‐June and mid‐July, depending on the latitude. It is both a diurnal and crepuscular species as it flies during daytime hours (Rojas et al., 2015), but shows mate searching flying activity between ∼5:00 p.m. and 10:00 p.m. with mating activity that can extend into the night (Nokelainen et al., 2012; Gordon et al. 2015) and a mating peak in laboratory around sunset (∼10:00 p.m. to 11:00 p.m.) (pers. obs.). Under laboratory conditions, it can produce up to three generations per year.

### MOTHS REARING AND STOCK MAINTENANCE

The laboratory stock was established in 2013 at the Department of Biological and Environmental Science, University of Jyväskylä (Finland) and new individuals were introduced yearly to the stock to maintain the genetic variability. During the stock maintenance, individual females were offered one randomly selected male to ensure offspring's paternity. Greenhouse temperature roughly followed the outdoor temperature (20–25°C) and natural light. Individuals were paired in 13 × 7 × 9 cm (*h* × *w* × *l*) transparent plastic boxes with mesh on the lid. Each box was provided with a small piece of moistened paper, where the moths could drink, and to offer a substrate for later oviposition. Three genotype lines have been established in the stock for experimental purposes, each one composed by numerous families. To avoid high inbreeding coefficient that could affect the moths’ survival and the experimental results, controlled matings are performed in each generation to ensure the most variable genotype‐family combination. The life‐history traits (fecundity, fertility, hatching success, offspring survival, and mating success) analyzed in this article come from 19 generations (i.e., 7 years) of data collection. Because mating pairs for the stock maintenance are not individually observed for successful copulation events, we followed a subset of these matings to determine whether heterozygote individuals have higher probability of copulating. These same individuals were then used to test for the individual's longevity. We introduce here the terminology used in the following sections; at the precopulatory stage, we use “copulation probability” to define the likelihood of the paired individuals to copulate; at the postcopulatory stage, we use “reproductive output” when referring to fecundity, fertility, and hatching success, and “mating success” to refer to the likelihood of reproductive failure. Finally, throughout this work, when referring to “genotype,” we refer to the sire or dam's genotype.

### PRECOPULATORY STAGE: COPULATION PROBABILITY AND MATING DELAY

We followed a total of 292 pairs, of which 180 were white (87 WW and 61 Wy genotypes) and 112 were yellow (yy) males. Among females, 73 were WW, 53 Wy, and 89 yy (see the Supporting Information for the complete crossing scheme). Each male was paired with a single female. Pairs were set at 4:00 p.m. and observed until midnight, approximately 1 hour after sunset when moths were not active anymore. All moths were 1–7 days old. We considered a mating to be successful if the mating pair was successfully formed within the 8 hours of observation. Otherwise, we considered it as not successful. We recorded the copulation success of each pair and the time it took to start mating (henceforth “mating delay”).

### POSTCOPULATORY STAGE: REPRODUCTIVE OUTPUT

To test the reproductive output of the different genotypes, we compared the fecundity (number of eggs), fertility (number of hatched larvae), and hatching success over 19 generations (i.e., 7 years) of life‐history trait data collected during routine maintenance of the common garden stock population. Because individuals had been reared in the greenhouse for several generations, we controlled for the effect of inbreeding coefficient by adding it as fixed effect and tested its potential interactive effect with the genotype in the analyses of reproductive traits (see the Supporting Information for inbreeding coefficient calculation and Table S1). For each mating pair, the number of laid eggs was counted 4 days after the female had laid her first egg, and larvae were counted 14 days after the first one had hatched. The hatching success was calculated as the total number of larvae that hatched divided by the total number of eggs the female had laid. Larvae were divided to groups of 30, 14 days after hatching. This counting gives us an indication of the genotype's survival. We also tested for genotype differences in oviposition day and hatching day (i.e., the number of days it took for each individual to, respectively, lay the first egg or for the first larvae to hatch). A total of 2714 genotyped individuals were used for these analyses, of which 1566 were sires (111 WW, 522 Wy, and 933 yy) and 1148 were dams (150 WW, 351 Wy, and 647 yy).

### POSTCOPULATORY STAGE: MATING SUCCESS

Because the life‐history trait data collected during stock maintenance mainly take into account successful matings and thus represent fitness after selection, it is important to separately analyze those who failed either to mate or produce viable offspring. Because the lack of offspring also translates in the lack of full known genotype, we classified individuals either as having a W (either WW or Wy genotype) or a y (i.e., yy genotype) allele. We identified three stages of failure: no eggs laid (i.e., no eggs), eggs were laid but no larvae hatched (i.e., egg hatching), and larvae hatched but none reached adulthood (i.e., adult eclosion). A total of 1059 matings (out of 2357 set) were considered unsuccessful (44.9%) with 1568 individuals and 561 pairs included in the analyses.

### LONGEVITY OF GENOTYPES

To follow individual longevity but avoid multiple matings, we removed the male from the mating box at about 1:00 p.m. the day after the mating and kept them in separated jars to follow their longevity.

### STATISTICAL ANALYSES

**Figure 2:**
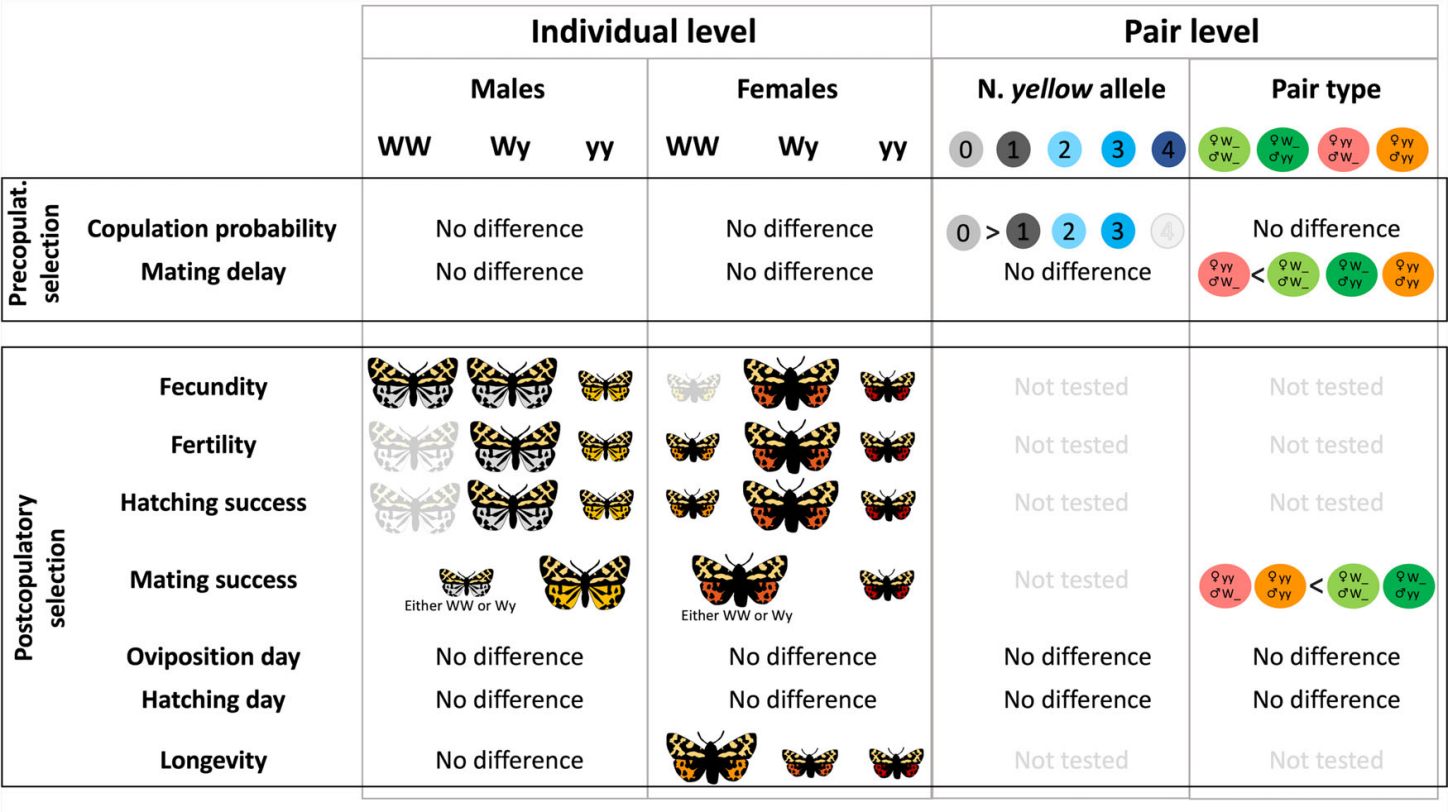
Summary of the effect of the genotype and alleles on the traits included in the analyzes. “No difference” refers to no effect of the genotype/alleles on the denoted trait. Full‐colored moths/dots indicate that the corresponding genotype/alleles plays a role on the denoted trait, and the size of the moth indicates the higher or lower trait output in genotype comparison. Grayscale moths/dots indicate no significant effect of such genotype/allele; however, the size hints at the higher or lower trend on such trait.

All analyses were performed in Rstudio (version 1.4.1717) (R Core Team 2013). The effect of individual full‐allele combinations (i.e., genotype) was tested both at the pre‐ and postcopulatory stage. Because several traits showed a general disadvantage of the yy genotype at the postcopulatory stage, we tested the effect of the y allele at the pair level. We classified the pairs either based on the number of y alleles in the pair (henceforth “number of y allele,” from 0 when both individuals are WW, to 4 when both are yy) or based on individuals that either had one W allele or both yy alleles (henceforth “pair type”). This allowed to test, respectively, for the effect of the y allele regardless of, or considering, the sex of the moth (see Table S2 for the sample size and spelled‐out pair classification, and Fig. 2 for a summary of the results).

#### Moth weight and age

Because mate choice and mating success can be affected by size and age, we tested whether genotype differences existed among the moths used at the precopulatory (weight and age) and postcopulatory stage (weight only) by fitting linear models with either “weight” or “age” as response variables and male or female genotype as fixed effects using the “lm” function (“stats” package version 4.1.1). We compared the mean weight and age between genotypes with *F*‐tests implemented with the “aov” function (“stats” package) and performed pairwise post hoc comparisons by estimated least‐square means using the “lsmeans” function (Tukey HSD adjustment; “lsmeans” package version 2.30‐0).

#### Precopulatory stage: Copulation probability and mating delay

Copulation event was recorded as a binary variable: 1 if the pair formed, 0 otherwise. At the individual level, we first tested for differences in the copulation probability by setting two Generalized Linear Models (GLMs) (one for males and one for females) with “copulation probability” as response variable, modeled with binomial distribution, and genotype, weight, their interaction, and age as fixed factors. We included the interaction between genotype and weight because of significant differences in weight between genotypes (reported later). We tested the overall effects of the variables with Chi‐square test implemented with the “anova” function.

We then analyzed the mating delay. Across years, the trials were performed by using moths reared in the three different generations, thus carried out in slightly different seasonal time. Because they mate preferentially 1–2 hours before the sunset (pers. obs.) and the sunset time is ∼9:30 p.m. in the first and third generations, and ∼11:00 p.m. during the second generation, we first tested whether the mating delay (response variable) was significantly affected by the generation time (fixed factor) and controlled for the effect of the year (random effect) with a Cox Proportional Hazard Model (henceforth “Cox model”) (function “coxph,” “survival” package, version 3.2‐11). Because the mating delay was significantly affected by generation (*χ* ^2^ = 143.14, df = 2, *P* ≤ 2.2 × 10^–16^) with the second generation (mean ± SE = 334 ± 20 min) leading to higher mating delay compared to the first (mean ± SE = 262 ± 11 min; estimated marginal means = –0.726 ± 0.111, *z* = –6.552, *P* ≤ 0.0001) and the third (mean ± SE = 246 ± 11 min) generations (estimated marginal means = –0.905 ± 0.216, *z*‐ratio = –3.67, *P*‐value = 0.0001), we standardized the mating delay to make it comparable for later analyses by centering the mean (mean = 0 and SD = 1). We tested the effect of genotype, weight, age, and generation (fixed effects) on mating delay (response variable) with two Cox models: one for males and one for females. The male model included the interactions “genotype x generation,” “genotype x weight,” and age as fixed effects, whereas the female model included the interaction genotype by weight, generation, weight, and age. We did not fit “genotype × generation” interactions because we did not test WW females in the second generation. We then tested for the effect of “number of y allele” and “pair type” on copulation probability (response variable 1) and mating delay (response variable 2) by fitting two GLMs with binomial responses (for response variable 1) and two Cox models (for response variable 2).

#### Postcopulatory stage: Reproductive output

To test for differences in the number of eggs, larvae, and hatching success (response variables), we fit four Generalized Linear Mixed Models (GLMMs) per response variable: two with Poisson distribution and two with negative binomial distribution, of which two accounted for zero inflated distribution (“glmmTMB” function from “glmmTMB” package version 1.1.3). We included genotype, weight, inbreeding coefficient, and two interactions (“genotype × weight” and “genotype × inbreeding coefficient”) as fixed effects and family as random effect to control for the effect of relatedness. We standardized both the weight and the inbreeding coefficient variables (by centering the mean and SD = 1) to include them in the interaction with a discrete variable (the genotype). The model with the lowest AIC value was selected as the best (Table S8, Panel a). For all three response variables, we used type III analyses of variance to test for the effect of the interactions on the response variable, and if the effects were not significant (*P* > 0.05), we removed the interactions from the final model. Finally, we performed genotype pairwise comparisons based on estimated marginal means (“emmeans” function of the “emmeans” package, version 1.7.2).

In addition, by considering pairs with only one WW and one yy individual (which ensures Wy offspring), we tested whether the heterozygote advantage could come from the dam or sire's side. We thus tested whether fecundity, fertility, and hatching success (three response variables) differed between pairs (fixed factor) by fitting two GLMs per response variable, one with a Poisson and the other with a negative binomial distribution. We chose the models with negative binomial distribution due to their lower AIC (Table S3).

Finally, we tested for the effect of genotype, “number of y allele,” and “pair type” (fixed factors) on the number of days both to lay eggs (response variable “oviposition day”) and for the eggs to hatch (response variable “hatching day”) by setting two GLMMs per response variable, one with Poisson and one with negative binomial distribution, genotype as fixed factor and generation as random factor, and four GLMs with the same response variables and distribution, but either “number y allele” or “pair type” as fixed effects. GLMs with Poisson distribution were chosen because of their lower AICs (Table S4).

#### Postcopulatory stage: Mating success

To test for differences in mating success between the W and y allele (fixed factor) and “pair type” (fixed factor), we fit four GLMMs, one to test for the allele effect regardless of sex, two models considering moth sex (one for males and one for females), and the final one for the pair effect. In all the four models, we determined the probability of successfully mating (response variable) by the count of successful over the unsuccessful matings through the “cbind” function and modeled with binomial distribution. We set generation as a random effect and used the function “weights” to specify the total number of matings that were set per generation. We tested the effect of the “pair type” using pairwise comparisons based on estimated marginal means (Tukey HSD adjustment).

#### Longevity of genotypes

To test whether longevity differed between genotypes, we fit two Cox models, one for males and one for females, with individuals’ life span (days) as response variable, and genotype as fixed factor.

## Results

### MOTHS WEIGHT AND AGE

Genotype did not affect male (*F*_(2;1199)_ = 2.567, *P* = 0.077) or female (*F*_(2;938)_ = 0.246, *P* = 0.782) weight of the individuals used in the postcopulatory analyses, but did for those used at the precopulatory stage. In both sexes, WW individuals were significantly heavier than yy individuals (estimated marginal means; males = 14.10 ± 3.39, *t* = 4.162, *P* = 0.0001; females = 16.40 ± 6.18, *t* = 2.652, *P* = 0.0233), and WW females were also heavier compared to Wy females (estimated marginal means = 23.69 ± 7.03, *t* = 3.371, *P* = 0.0026). Age did not differ between male (*F*_(2,257)_ = 0.898, *P* = 0.409) or female genotypes (*F*_(2,212)_ = 1.357, *P* = 0.26).

### PRECOPULATORY STAGE: COPULATION PROBABILITY AND MATING DELAY

Although we found genotype‐specific differences in weight, the copulation probability in either sex was not affected by their interaction (males: genotype × weight = *χ* ^2^_(2,250)_ = 5.5438, *P* = 0.0625; females: genotype × weight = *χ* ^2^_(2,205)_ = 2.4382, *P* = 0.2955). Copulation probability was not affected by male or female genotype, male weight, or male and female age (Table S5, Panel a). Interestingly, the heavier the female, the lower the copulation probability (GLM; Estimate = –0.4776 ± 0.1538, *z* = –3.106, *P* = 0.0019). The mating delay was significantly affected by the generation, suggesting that environmental cues (e.g., the sunset/light) may influence the mating behavior (Table S5, Panel b). Males took significantly longer in the second generation compared to the first and third (coxph; second vs. first; exp(coef) = 2.1212 ± 0.2739, *z* = 2.745, *P* = 0.0060; second vs. third; exp(coef) = 2.7969 ± 0.2884, *z* = 3.567, *P* = 0.0004), whereas females took significantly longer only compared to the third generation (coxph; second vs. third; exp(coef) = 3.0448 ± 0.3285, *z* = 3.389, *P* = 0.0007). Besides the effect of the environmental cues, no other traits played a significant effect on the mating delay. These include the lack of interaction between male genotype and generation (LR test; *χ* ^2^ = 0.8738, df = 2, *P* = 0.6460), the lack of genotype‐specific effect of weight (LR test; male genotype × weight: *χ* ^2^ = 3.1702, df = 2, *P* = 0.2049; female genotype × weight: *χ* ^2^ = 1.5839, df = 2, *P* = 0.4530), and lack of significant effect of genotype, weight or age, both in males and females (Table S5, Panel b).

Although there was no precopulatory selection at the individual level, a closer look at the copulation probability and mating delay suggests that the allele combination may play an indirect role in these traits, at least for some genotypes. The number of y alleles in the mating pair significantly affected the copulation probability (*χ* ^2^ = 12.996, df = 4, *P* = 0.0113), where pairs with zero y alleles had a higher copulation probability in general, and significantly higher than pairs with one, two, and three y alleles (Table S6). We found, however, no significant effect of the pair type (*χ* ^2^ = 3.6337, df = 3, *P* = 0.3038) on the copulation probability, suggesting a general effect of the allele combinations on the mating success rather than sex‐specific contribution. For the mating delay, we found somewhat the opposite pattern, as it did not differ according to the number of y alleles (LR test; *χ* ^2^ = 2.26, df = 4, *P* = 0.6872) but the “femyy + maleW‐allele” pair type mated significantly faster than all the other pair types (Table S7). Genotype‐specific advantages might be relative to the mating partner and thus can arise at the pair level.

### POSTCOPULATORY STAGE: REPRODUCTIVE OUTPUT

For the six final models selected, the lowest AICs were given by the zero inflated with negative binomial models (Table S8, Panel a). Neither interactions (“genotype × weight” and “genotype × inbreeding coefficient”) were significant and were excluded from the final models (Table S8, Panels b–d). This suggests that weight and inbreeding coefficient did not affect the reproductive output in a genotype‐specific manner, despite, for example, genotype‐specific differences in the inbreeding coefficient. Although the genotype did not explain the mean differences in fecundity, fertility, and hatching success (Table S9 [Panels b and d], Table S10 [Panel b], Table S11 [Panel b], and Table 1 [Panels a and c]), it had a strong effect on the probability of reproductive failure. This suggests that genotypes differ in their likelihood of reproductive failure rather than the number of eggs, larvae, or proportion of eggs hatched.

**Figure 3:**
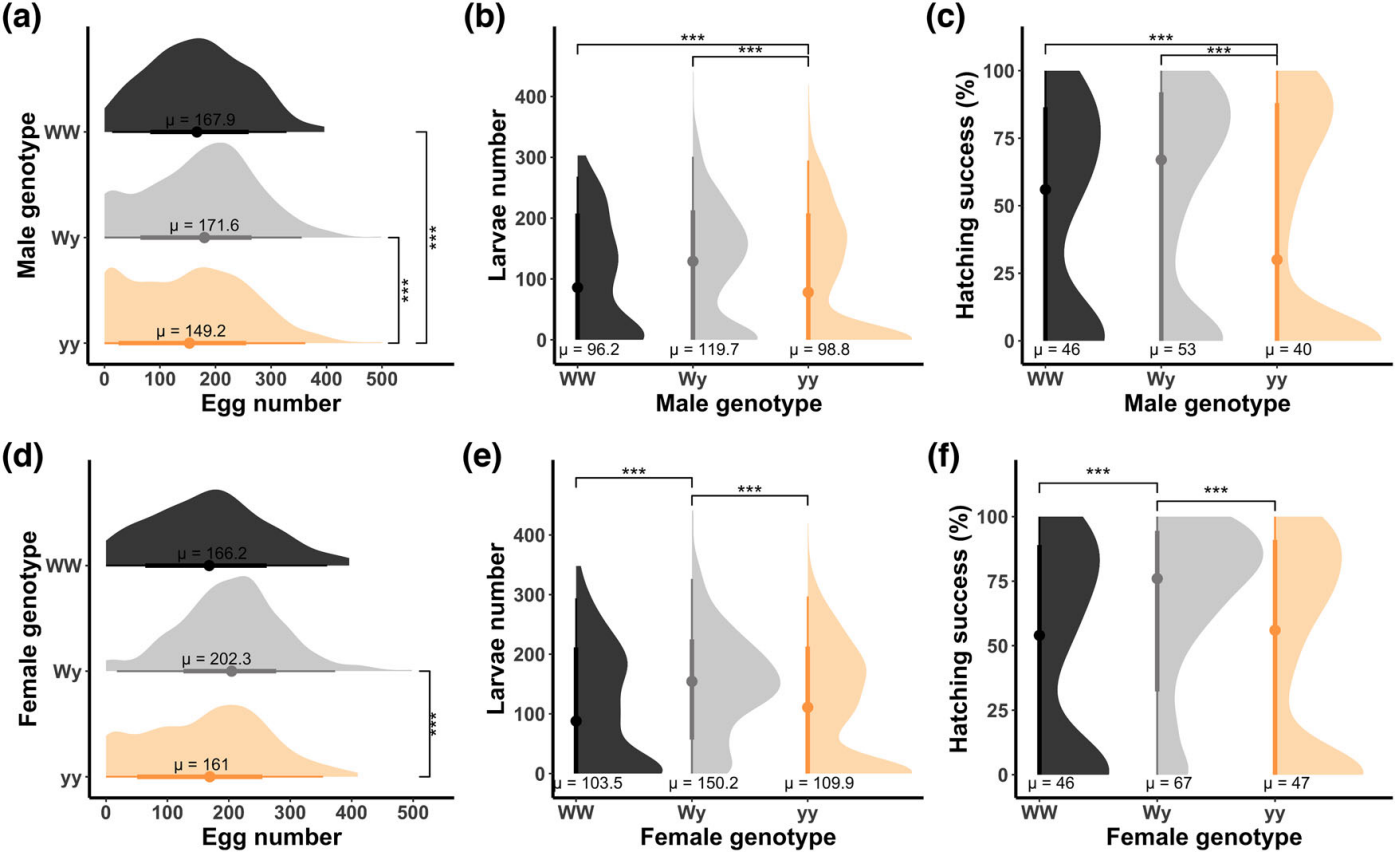
The graph illustrates differences in the fecundity, fertility, and hatching success between genotypes, in males (top row) and females (bottom row). Statistically significant differences are marked with asterisks.

Genotype, female weight, and inbreeding coefficient had a significant effect on the fecundity trait (Table S9, Panel a). yy males had significantly fewer eggs (mean ± SE = 149.2 ± 3.7) compared to WW (mean ± SE = 167.9 ± 8.3) and Wy (mean ± SE = 171.6 ± 4.9) males (Table S9, Panel c; Fig. 3a). yy females laid a significantly lower number of eggs (mean ± SE = 161 ± 4.2) compared to Wy females (mean ± SE = 202.3 ± 5) but not compared to WW females (mean ± SE = 166.2 ± 7.8) (Table S9, Panels d and e; Fig. 2d). The yy genotype disadvantage was due to both a lower egg count and a higher probability of failing to have eggs at all, both in males and females (Table S9, Panels c and e). Weight had a significant effect in females (Table S9, Panel a) with the heavier the female, the higher the number of eggs laid (Table S9, Panel e), whereas no significant effect of the weight was detected for males (Table S9, Panel a). Weight had a significant effect on the count (number) of eggs laid but did not affect the probability of zero count (Table S9, Panel e). No interaction between inbreeding coefficient and genotype was detected but the inbreeding coefficient had a significant effect on the number of eggs laid (Table S9, Panel a), with the higher its value, the lower the egg count (Table S9, Panels c and e). Interestingly, this did not affect the probability of having zero eggs (Table S9, Panels c and e).

Wy females had a significantly lower probability of egg hatching (i.e., having larvae) failure (Table 1, Panel b; Fig. 3e). This was not repeated in males, as yy males had lower probability of having larvae than WW and Wy males (Table S10, Panel b; Fig. 3b). The significant differences were in the probabilities of failure (zeroes) and not in the number (count) of larvae. Therefore, the female Wy advantage is due to the significantly lower probability in failing to have larvae at all compared to the other two genotypes (Table 1, Panel b). The effect of female weight on fertility was significant (Table S10, Panel a) with the heavier the female, the higher the number of larvae that hatched (Table 1, Panel b). This was not seen in males (Table S10, Panel c). The inbreeding coefficient had a significant effect on the fertility trait (Table S10 [Panel c] and Table 1 [Panel b]), where the higher the inbreeding coefficient, the lower the number (count) of larvae in males only (Table S10, Panel c) but not in females (Table 1, Panel b). In addition, the higher the inbreeding coefficient, the higher the probability of zero larva both in males and in females (Table S10 [Panel c] and Table 1 [Panel b]).

**Table 1:** Panels (a) and (c) report female genotype pairwise comparisons for the fertility and hatching success traits. Panels (b) and (d) report the GLMM output for, respectively, the fertility and hatching success trait in females

Fertility:					
(a) Pairwise comparisons based on estimated marginal means; Tukey HSD adjustment					
Contrast	Estimate	SE	df	*t*	*P*
Wy‐WW	0.074	0.082	925	0.894	0.644
Wy‐yy	0.035	0.052	925	0.674	0.779
WW‐yy	–0.039	0.078	925	–0.495	0.874
(b) Zero inflated; Intercept = Wy genotype					
	Estimate	SE	*z*	*P*	
Count model					
Intercept	5.054	0.040	125.31	**<2 × 10^–16^**	
WW genotype	–0.073	0.082	–0.89	0.371	
yy genotype	–0.035	0.052	–0.67	0.500	
Weight	0.217	0.026	8.47	**<2 × 10^–16^**	
Inbreeding coefficient	–0.010	0.027	–0.39	0.699	
Zero inflated model					
Intercept	–2.456	0.229	–10.721	**<2 × 10^–16^**	
WW genotype	1.225	0.310	3.946	**7.95 × 10^–5^**	
yy genotype	1.566	0.249	6.293	**3.12 × 10^–10^**	
Weight	–0.026	0.087	–0.300	0.764	
Inbreeding coefficient	0.432	0.092	4.682	**2.84 × 10^–6^**	
Hatching success:					
(c) Pairwise comparisons based on estimated marginal means; Tukey HSD adjustment					
Contrast	Estimate	SE	df	*t*	*P*
Wy‐WW	0.089	0.068	834	1.324	0.382
Wy‐yy	0.041	0.044	834	0.933	0.619
WW‐yy	–0.048	0.064	834	–0.756	0.730
(d) Zero inflated; Intercept = Wy genotype					
	Estimate	Std. Error	*z*	*P*	
Count model					
Intercept	4.284	0.034	126.06	**<2 × 10^–16^**	
WW genotype	–0.089	0.068	–1.32	0.185	
yy genotype	–0.041	0.044	–0.93	0.351	
Weight	0.0004	0.021	0.02	0.986	
Inbreeding coefficient	0.005	0.023	0.23	0.817	
Zero inflated model					
Intercept	–2.350	0.230	–10.215	**<2 × 10^–16^**	
WW genotype	1.210	0.312	3.876	**0.0001**	
yy genotype	1.559	0.251	6.216	**5.12 × 10^–10^**	
Weight	–0.005	0.089	–0.060	0.952	
Inbreeding coefficient	0.382	0.095	4.003	**6.25 × 10^–5^**	

The hatching success was significantly affected by the individual genotype (Table S11 [Panel c] and Table 1 [Panel d]), with Wy females having a higher likelihood of hatching success compared to the other two genotypes (WW mean ± SE = 0.46 ± 0.03, Wy mean ± SE = 0.67 ± 0.02, yy mean ± SE = 0.47 ± 0.02; Table 1, Panel d; Fig. 3f). In males, the yy genotype had a lower likelihood of hatching success than the other two genotypes (WW mean ± SE = 0.46 ± 0.04, Wy mean ± SE = 0.53 ± 0.02, yy mean ± SE = 0.40 ± 0.01; Table S11, Panel c, Fig. 3c). We found, therefore, strong female heterozygote advantage in fertility and hatching success expressed in their higher likelihood of having larvae and higher likelihood of hatching success. Weight had a significant effect in males but not in females (Table S11, Panel a). Interestingly, the heavier the male, the lower the probability of hatching success (Table S11, Panel c). The inbreeding coefficient significantly affected males and females (Table S11 [Panel c] and Table 1 [Panel d]) with lower probability of hatching success as its value increases.

Finally, the Wy advantage does not seem to be due to either maternal or paternal effect. The number of eggs (glm.nb; estimate = 0.031 ± 0.127, *z* = 0.24, *P* = 0.81), larvae (glm.nb; estimate = –0.066 ± 0.175, *z* = –0.377, *P* = 0.706), or the hatching success (glm.nb; estimate = 0.164 ± 0.288, *z* = 0.589, *P* = 0.556) did not differ between pairs where either the dam or the sire was WW and the other yy. This suggests that the higher Wy fitness is due to the allele combination (W and y) per se, rather than being determined by the dam or sire's side. We found no differences based on the individual genotype or due to the effect of the pair for the oviposition day and hatching day (Table S4) suggesting no particular effect of the color locus on these traits.

### POSTCOPULATORY STAGE: MATING SUCCESS

**Figure 4:**
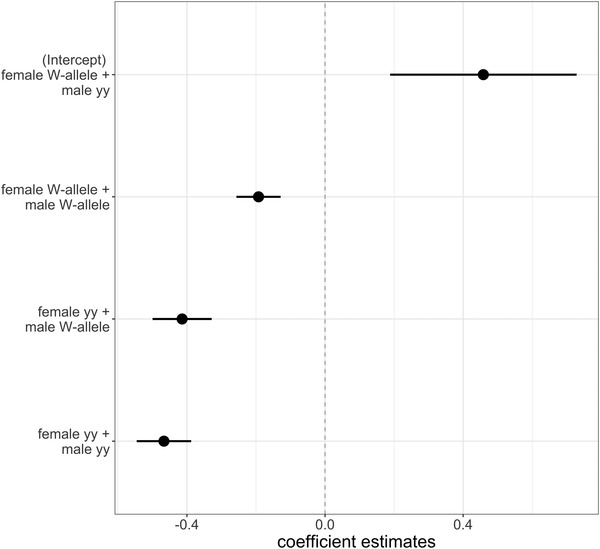
Coefficient estimates of the probability of pair type's mating success. Except for the nonsignificant difference between “female yy + male yy” and “female yy + male W‐allele,” all the other pairwise comparisons were significantly different (see Table S12 for a reference).

With 78% of the unsuccessful matings having eggs and larvae, the mating failure is more likely to take place at the postcopulatory rather than precopulatory stage. The most sensitive stage seems to be the egg‐hatching stage (62%), which was significantly higher than matings that had no eggs (23%; *χ* ^2^ = 17.89, df = 1, *P* = 2.335 × 10^–5^) and than matings that had no adult eclosing (15%; *χ* ^2^ = 28.69, df = 1, *P* = 8.502 × 10^–8^). No differences were found between the no‐egg and adult‐eclosing stage (*χ* ^2^ = 1.68, df = 1, *P* = 0.19). There was no effect of either the sire or the dam at the different stage levels (no‐eggs stage, *χ* ^2^ = 0.56, df = 1, *P* = 0.46; egg‐hatching stage, *χ* ^2^ = 0.072, df = 1, *P* = 0.79; adult‐eclosing stage, *χ* ^2^ = 0.13, df = 1, *P* = 0.72), suggesting no sex‐specific cause of failure. Y‐allele individuals (i.e., yy genotype) had a significantly higher probability of failing to have offspring than W allele individuals (W vs. y; estimate = –0.075 ± 0.007, *z* = –10.475, *P* ≤ 2 × 10^–16^). These results were likely influenced by females, as y allele females failed significantly more than W allele females (W vs. y females; estimate = –0.272 ± 0.020, *z* = –13.38, *P* ≤ 2 × 10^–16^), whereas y allele males had significantly higher probability of succeeding in having offspring compared to W allele males (W vs. y males; 0.044 ± 0.011, *z* = 4.014, *P* = 5.97 × 10^–5^). The generation effect accounted in average for 15% of the variation in the probability of failing (16% in females and 13% in males). At the pair level, “female yy + male yy” and “female yy + male W‐allele” pair types had the lowest probability of having offspring (estimated marginal means = 0.0527 ± 0.0426, *z* = 1.239, *P* = 0.6023), whereas the probability of failing significantly differed between all the other pair comparisons (*P*‐values < 0.05) (Table S12; Fig. 4).

### LONGEVITY OF GENOTYPES

All males’ genotypes had similar life spans after mating once (LR test; males; *χ* ^2^ = 0.297, df = 2, *P* = 0.862), whereas WW females lived significantly longer than the other two female genotypes (coxph; WW vs. Wy; exp(coeff) = 2.2614 ± 0.3653, *z* = 2.234, *P* = 0.0255; WW vs. yy; exp(coeff) = 1.99 ± 0.3192, *z* = 2.156, *P* = 0.0311). For a summary of these results, see Figure 2.

## Discussion

We investigated the effect of color alleles and genotypes from pre‐ to postcopulatory stage in maintaining warning color polymorphism within wood tiger moth populations. Carrying one or two copies of the yellow allele affected the reproductive fitness in a stage‐specific way, from higher likelihood of reproductive output when females carry one copy of the allele (i.e., heterozygote advantage), to lower likelihood of reproductive output success and lower mating success when individuals carry two copies. Thus, the *yellow* allele might have a pleiotropic effect on several life‐history traits that can contribute to the maintenance of polymorphism in male coloration. Although we found little contribution of male genotype across the reproductive sequence, female genotype had a significant effect, especially for reproductive success, and likely therefore contributes to the persistence of polymorphism in male coloration. Although all the genotypes, regardless of the sex, had an equal copulation probability and mating delay, Wy females had higher reproductive output (fertility and hatching success) and thus higher offspring survival. Pairs with yy females had shorter mating delay and were more likely to fail in having any offspring. The presence of the *yellow* allele affected the fitness both at the individual and pair level, such as a lower reproductive output in males, and across different steps of the reproductive process for females. Our results thus show the role of genotype‐dependent female reproductive success in maintaining male hindwing coloration. Overall, these results suggest that the color locus is pleiotropic with a number of life‐history traits, allowing for the maintenance of within‐species phenotypic diversity.

### WEAK EFFECT OF PRECOPULATORY SELECTION

At the precopulatory stage, 43% of paired individuals did not copulate suggesting some form of female or male rejection. The lack of copulation probability and mating delay differences between genotypes suggests that precopulatory selection may be a weak selective force on the genotypes and, at this stage, neither males nor females can avoid mating with partners with lower fitness prospects. These results are in accordance with a previous study with a similar mating experiment setup that showed equal mating probability between white and yellow phenotypes (Chargé et al., 2016). The hypothesis that sexual selection is more likely to take place after the copulation event, rather than resulting from precopulatory selection, may be further supported by the low (23%) percentage of failed matings that did not have eggs, a proxy for the lack of copulation event. However, we cannot exclude that the lack of differences in the copulation probability may have been masked by a trade‐off between securing at least one mating (and therefore some offspring) and exerting mate choice (see Kokko and Mappes 2005).

Females of different species have been shown to exert stronger sexual selection when presented with a choice (Dougherty and Shuker, 2015). Virgin females, due to the uncertainty of finding a second mate and the risk of dying unmated, are expected to be less choosy and may accept to mating randomly if they fear no further male will be encountered (Kokko and Mappes, 2005; Dougherty and Shuker, 2015). In addition, individuals may get choosier in later matings (Kokko and Mappes 2005; Gao et al., 2020), which might explain the lack of differences in copulation probability. This explanation may also be supported by the lack of differences in the mating delay; if any choice were to be made based on some trait, it might have been translated into a different mating delay. Instead, the mating delay was higher in the second generation because this species is mostly sexually active around sunset (pers. obs.), which is about 2 hours later than the first and third generations (∼11:30 p.m. vs. ∼9:30 p.m.). Other studies on the species have shown that differences in male copulation probability, and particularly the white male advantage, may be condition dependent (stress‐induced condition; Nokelainen et al., 2012), due to the effect of white mixed‐lineage advantage (more heterozygous individuals; Gordon et al., 2018), or context dependent, in which the most common morph has higher mating success (Gordon et al., 2015). Mating differences, or lack thereof, in the wood tiger moth may be, therefore, determined by the ecological context or be based on a different trait (e.g., the sex pheromone).

### HETEROZYGOTE ADVANTAGE FOR THE MAINTENANCE OF COLOR POLYMORPHISM

At the postcopulatory stage, we found a significant effect of the genotype on fecundity, fertility, and hatching success. In particular, heterozygote (Wy) females had higher likelihood of fertility, offspring survival, and hatching success than the other two genotypes, suggesting that male hindwing coloration is maintained by a rather strong heterozygote advantage effect. The Wy advantage does not seem to be due to either dam or sire's effect (i.e., Ww × yy pairs do not show differences in their reproductive output) or due to differences in oviposition or hatching strategies, suggesting that the heterozygote advantage is a consequence of the W and y allele combination. Wy females had, indeed, a significantly lower probability of zero fertility, which translated into higher hatching success than both the homozygotes. Our results add to a few other known cases of heterozygote advantage (*Buteo buteo*, Krüger et al., 2001; *wolves*, Hedrick et al., 2014; *Heliconius numata*, Jay et al., 2021). The advantage of the dominant (W) allele in our species does not appear to change for fitness‐related measures supported by the general advantage of Wy (and WW genotype) and over the general disadvantage of the yy genotype throughout the reproductive output, a pattern somewhat opposite to the wolf of the Yellowstone National Park (Coulson et al., 2011; Hedrick et al., 2014). In contrast, the heterozygosity advantage in the wood tiger moth may be context dependent: in mating probability either due to female choice or intrasexual competition (Gordon et al., 2018), in the reproductive output (this study), or as defense against predators (Winters et al., 2021), which suggests the importance of considering both natural and sexual selective processes.

### PLEIOTROPIC EFFECT OF THE YELLOW ALLELE

The presence of one or two copies of the *yellow* allele affected several steps of the reproductive sequence, from copulatory probability, to mating delay, reproductive output, and mating success, especially in females. Females carrying one *yellow* allele (i.e., Wy) had higher reproductive output than the other two genotypes (i.e., heterozygote advantage). The W and y allele combination might therefore lead to a genetic compatibility advantage that give rise to increased offspring survival, and higher likelihood of hatched eggs (i.e., hatching success). Bearing two copies of the *yellow* allele affected other traits of the reproductive sequence, such as copulation probability, mating delay, and mating success of pairs with yy females. About 55% of the pairs with yy females copulated (against, e.g., 80% of WW × WW pairs), whereas pairs with yy females and white (i.e., WW or Wy) males copulated faster than the other pair types. WW and Wy males have higher reproductive output than yy males, which might be a reason why yy females are more willing to accept white males compared to the yy males. Females carrying two copies of the *yellow* allele had a higher likelihood of reproductive failure regardless of the male they mated with. Mating with a yy female may thus be particularly costly to males.

We thus suggest that genotypic differences in life‐history traits are likely due to pleiotropic effects of the *yellow* allele, especially because these effects are expressed in females without the yellow phenotype. The pleiotropic effect of the *yellow* allele extends to males as well. The male yellow coloration confers better protection against predators (Nokelainen et al., 2012, 2014; Rojas et al., 2017), but there are trade‐offs with the mating probability (Nokelainen et al., 2012), the reproductive output (Gordon et al., 2018; this study), and their ability to disperse (Gordon et al., unpublished). Recent examples of the pleiotropic effect of color loci on life‐history traits have been found in the warningly colored seed bug (*Lygaeus simulans*) (Balfour et al., 2018) and *Heliconius numata* (Jay et al., 2021).

The male coloration in the wood tiger moth is likely regulated by a *yellow*‐family gene (Brien et al., 2022), which is conserved across insects (Ferguson et al., 2011) and has well‐known functions in the melanin production pathway (Wittkopp et al., 2002). *Yellow* genes have also been shown to have pleiotropic effects on life‐history and behavioral traits (Bastock, 1956; Massey et al., 2019; Connahs et al., 2021). Loss of the *yellow* gene function in *D. melanogaster* results in reduced mating success due to changes in the courtship behavior (Bastock, 1956) and to structural changes in the sex combs used to grasp the female (Massey et al., 2019). The *yellow* gene has the opposite effect in *Bicyclus anynana* where its expression needs to be suppressed for the males to properly express courtship behavior (Connahs et al., 2021). Thus, we suspect that the *yellow* locus is influencing life‐history traits as well as wing coloration also in the wood tiger moth, although the exact genetic mechanism is yet unknown.

### GENERAL IMPLICATIONS FOR THE MAINTENANCE OF THE COLOR POLYMORPHISM

Across its distribution, the wood tiger moth shows a striking level of phenotypic diversity, both across and within populations (Hegna et al., 2015). From our results, the overall advantage of individuals bearing at least one dominant W allele, and the pleiotropic effect of the *yellow* allele, could theoretically explain populations that are naturally W male biased, such as the Finnish population. However, the 2:1 (white:yellow) ratio expected by the dominant W allele advantage is hardly found in natural populations, even in the light of the higher likelihood of y‐bearing individuals to show disadvantage along the reproductive sequence. This suggests that other mechanisms and selective forces are at play. The extensive literature on this study system shows indeed that male morphs experience a multitude of morph‐specific selective pressures, from predation (Nokelainen et al., 2012, 2014; Rojas et al., 2017, Winters et al., 2021) linked also to light environment (Nokelainen et al., 2022a), to immune response (Nokelainen et al., 2013), and density‐dependent effects (Gordon et al., 2015). This likely affects the expected ratio of white and yellow morphs in natural populations. Future quantifications of the genotype frequencies of natural populations will shed more light on the mechanisms maintaining both alleles.

### PRE‐ AND POSTCOPULATORY EFFECT OF TRAITS BEYOND COLOR GENOTYPE

Mate choice and mating probability may also be based on size or age. However, no effect of age in either sex or male weight was found to affect the copulation probability and mating delay despite white males being heavier than yellow males (and in general, WW individuals being heavier than the other two genotypes). The lack of age and weight effect could be due to the lack of mate choice or adaptation to lab conditions. Although female weight did not play a role in mating delay, it was interesting to notice that the heavier the female, the lower her copulation probability. A similar result was found in azure damselflies males (*Coenagrion puella*) in which lighter males have higher mating success (Banks and Thompson, 1985). Banks and Thompson (1985) put forward the hypothesis that heavier males may be less active due to their bigger size, thus less likely to find a female. As in our experiment, mating trials were carried out in a confined space and females were more easily spotted by males than in a natural scenario, the lower copulation probability of heavier females may be due to between‐females behavioral differences. For instance, heavier wood tiger moth females may be more prone to actively reject males than lighter females. Although this hypothesis should be properly tested, it has been already shown that in Lepidoptera male harassment can be costly to females (Merrill et al., 2018) and females actively reject males to the point they can override male preference (Chouteau et al., 2017). This behavioral hypothesis is also in line with the lowest yy female mate acceptance toward males carrying the W allele that lacks the deleterious elements associated with the y allele when expressed in homozygote yy males (at least for the reproductive success).

At the reproductive output stage, female, and not male, weight played a significant role in fecundity and fertility, despite larger males produce bigger spermatophores (Chargé et al., 2016). Not surprisingly, heavier females laid more eggs. This is in accordance with Santostefano et al. (2018) and it is expected because this species is a capital breeder and females are born with all the eggs that can be potentially fertilized (Tammaru and Haukioja, 1996). Heavier females also had higher fertility. Therefore, female weight may be a trait that males could select for. It is interesting to notice that heavy males had lower hatching success. A previous study on the wood tiger moth (Santostefano et al., 2018) showed that mating with heavier males led to a lower number of eggs laid. Because heavier males produce bigger spermatophores (Chargé et al., 2016), a negative correlation between male weight and hatching success may be the result of trade‐offs; being heavy and therefore having invested more resources into mass development may trade‐off with the quality of the spermatophore, or heavier males may spend more energy than lighter males in finding and/or courting a female, therefore lowering the resources available for spermatophore production.

## Conclusion

Altogether these results suggest that wood tiger moth male coloration is maintained through stage‐specific color allele and genotype advantages across the reproductive sequence, from copulation probability to offspring survival. Although individuals do not seem to avoid mating with partners with lower fitness prospects, the strong female heterozygote advantage in fertility, hatching success, and offspring survival offers a powerful mechanism for both alleles to be maintained within the population. Male hindwing coloration seems also to be maintained through the pleiotropic effect of the *yellow* allele, which affects specific traits of the reproductive sequence, from shortening the mating delay, to being correlated with higher reproductive failure and in general, with the reproductive output. In nature, populations are typically exposed to complex ecological interactions, multiple mechanisms, and selective forces. Such multiple mechanisms concurrently interact and allow for life‐history trait variability maintenance through pleiotropy (this study, Mérot et al., 2020) and thus maintain complex color polymorphisms even in the situation when selection is positively frequency dependent (Gordon et al., 2015; Chouteau et al., 2016).

## AUTHOR CONTRIBUTIONS

CDP collected the precopulatory stage data, analyzed the data, and wrote this article. KS and JK created the genotype lines, reared numerous generations of moths, and collected the life‐history traits data. SG initiated data analyses. TK helped with the inbreeding coefficient analyses and contributed to data analysis. JM conceptualized and coordinated the work. SG, TK, and JM contributed to the writing of the article. All authors read and approved the final version of the manuscript.

## CONFLICT OF INTEREST

The authors declare no conflict of interest.

## DATA ARCHIVING

The data used in this study are accessible from the following repository: https://doi.org/10.5061/dryad.g1jwstqth. The data will be released after 6 months.

Associate Editor: M. Kronforst

Handling Editor: T. Chapman

## Supporting information


**Table Supp. info 1**. Table a) reports the model selection for testing if inbreeding coefficients differ at the genotype level, and genotype pairwise comparisons.
**Table Supp. Info 2**. Sample size of the individuals tested and all possible pair combinations used in the experiments.
**Table Supp. Info 3**. The table below reports the model selection for the analyzes that tested whether the heterozygote advantage was linked to the sire or dam's side.
**Table Supp. Info 4** The upper table below reports the model selection for the oviposition and hatching day considering both models at the individual level (males and females) and at the pair level (number of yellow alleles in the pair and the pair type).
**Table Supp. Info 5**. Table a) reports the effect of the genotype, weight and age on the mating probability, for males (upper part) and females (lower part) separately through Chi‐square test.
**Table Supp. Info 6**. The upper part of the table reports the effect of the number of *yellow* allele and the pair type on the copulation probability through LR‐test.
**Table Supp. Info 7**. The upper table reports the effect of the number of yellow allele and the pair type on the mating delay through LR‐test.
**Table Supp. Info 8**. Table a) reports the model selection for the fecundity, fertility and hatching success traits for males and females.
**Table Supp. Info 9**. Table a) reports the effect of genotype, weight and inbreeding coefficient on the fecundity of males and females.
**Table Supp. Info 10**. Table a) reports the effect of genotype, weight and inbreeding coefficient on the fertility of males and females.
**Table Supp. Info 11**. Table a) reports the effect of genotype, weight and inbreeding coefficient on the hatching success of males and females.
**Table Supp. Info 12**. The table reports the pairwise comparisons between pair type for the mating success.
