# Supplementary material for: Heterozygote advantage and pleiotropy contribute to intraspecific color trait variability
Source: Evolution. 2022 Aug 29;76(10):2389–403. doi: 10.1111/evo.14597 (PMC9805086; doi:10.1111/evo.14597)
Supplement: Supplementary file 1 — Table Supp. info 1. Table a) reports the model selection for testing if inbreeding coefficients differ at the genotype level, and genotype pairwise comparisons. Table Supp. Info 2. Sample size of the individuals tested and all possible pair combinations used in the experiments. Table Supp. Info 3. The table below reports the model selection for the analyzes that tested whether the heterozygote advantage was linked to the sire or dam's side. Table Supp. Info 4 The upper table below reports the model selection for the oviposition and hatching day considering both models at the individual level (males and females) and at the pair level (number of yellow alleles in the pair and the pair type). Table Supp. Info 5. Table a) reports the effect of the genotype, weight and age on the mating probability, for males (upper part) and females (lower part) separately through Chi‐square test. Table Supp. Info 6. The upper part of the table reports the effect of the number of yellow allele and the pair type on the copulation probability through LR‐test. Table Supp. Info 7. The upper table reports the effect of the number of yellow allele and the pair type on the mating delay through LR‐test. Table Supp. Info 8. Table a) reports the model selection for the fecundity, fertility and hatching success traits for males and females. Table Supp. Info 9. Table a) reports the effect of genotype, weight and inbreeding coefficient on the fecundity of males and females. Table Supp. Info 10. Table a) reports the effect of genotype, weight and inbreeding coefficient on the fertility of males and females. Table Supp. Info 11. Table a) reports the effect of genotype, weight and inbreeding coefficient on the hatching success of males and females. Table Supp. Info 12. The table reports the pairwise comparisons between pair type for the mating success. [file EVO-76-2389-s001.docx]

**SUPPORTING INFORMATION**

*Inbreeding coefficient calculation; methods, statistical analyzes, and results*

Because we used individuals that were reared in the greenhouse for several generations (and Lepidoptera are generally known to be quite sensitive to inbreeding depression (Roush 1986; Cacoyianni et al., 1995), we calculated the inbreeding coefficient of the individuals used in the reproductive output analysis, to be able to take into account possible biases in analysis arising from inbreeding. For each individual (n = 3037) we assigned both parents and grandparents (n = 3337). When the identity of the parent was unknown, it was assigned value of 0 (n = 138). We could not assign the parents for the wild-caught individuals (n = 123) included in the pedigree or due to mislabelling (n = 15). Individuals with inbreeding coefficient of 0 due to missing parental information were excluded from final analyzes. The inbreeding coefficient was calculated using the ‘makeA’ function (‘pedigree’ package, v. 1.4) in R Studio. Inbreeding coefficients were retrieved from the diagonal of the resulting A-matrix (inbreeding coefficient = value in diagonal - 1). The obtained inbreeding coefficients were then linked back to the respective individuals and used for further analyzes. We tested whether the inbreeding coefficient (response variable) was significantly different between genotypes (fixed factor) by setting GLMs, one with beta distribution (function ‘betareg’) and one with gamma distribution (link ‘identity’ function). When testing for between sexes (fixed factor) differences, we set three independent GLMs, one per genotype to test for differences between sexes in inbreeding coefficient (response variable). Each model was set with both ‘betareg’ and ‘gamma’ distribution, and the best model was selected through AIC (Table 1). Both distributions accept only values between 0 and 1, therefore we transformed the 0 values in 0.000000001 (but no 1 values to 0.999999999 as no individual had such coefficient).The significant effects of the genotype was tested through type II analyzes of variance (F-test) using the ‘Anova’ function (‘car’ package, v3.0-10) and post-hoc genotype pairwise comparisons based on estimated marginal means were performed through the function ‘emmeans’ (Tukey-HSD adjustment; ‘emmeans’ package v. 1.6.1). The inbreeding coefficient was significantly affected by the individual genotype (F_(2;2170)_ = 143.75, P = < 2.2e-16). Post-hoc analysis revealed that the WW genotype was the most inbred genotype (mean ± se = 0.15 ± 0.003) and its inbreeding coefficient significantly differed from the Wy genotype (mean ± se = 0.08 ± 0.002) and the yy genotype (mean ± se = 0.09 ± 0.001). Likewise, Wy and yy genotype significantly differ in their mean inbreeding coefficient (Table Supp. Info 1a). In addition, Wy females and yy males were less inbred than their counterparts whereas no differences were detected between WW males and females (Table Supp. Info 1b). For these reasons, the inbreeding coefficient was included as fixed effects in the reproductive output analyzes (see Main Text).

*Linking the inbreeding coefficient to the reproductive output results*

Due to selected matings, laboratory adaptations and lack of mate choice, lab-reared insects are likely to have higher inbreeding coefficients than in natural populations. This can heavily and negatively affect, in just a few generations, the fitness of Lepidoptera (Roush 1986; Cacoyianni et al., 1995). Not surprisingly, the inbreeding coefficient had a negative impact on all the traits considered for the reproductive output (fecundity, fertility and hatching success), with the higher the value, the higher the probability of failure or of a lower count. We cannot exclude the higher inbreeding coefficient of the WW genotype to be a consequence of the stock maintenance and our selective process due to the challenges of spotting this genotype accurately. However, differences in the inbreeding coefficient between genotypes may not be the ultimate explanation for the genotype-specific differences in fitness. This is because, despite a higher inbreeding coefficient for the WW genotype, WW females had the longest lifespan and WW individuals had a tendency of higher mating probability (80% of WWxWW pairs mated). In addition, despite very different inbreeding coefficients, the reproductive output of WW vs Wy males, and WW vs yy females did not differ. Finally, although yy females had higher inbreeding coefficient than their male counterpart, males and females showed to be equally affected in their reproductive output (see Inbreeding coefficient calculation paragraph).

**Table Supp. info 1.** Table a) reports the model selection for testing if inbreeding coefficients differ at the genotype level, and genotype pairwise comparisons. Table b) reports the model selection at the sex level analyzes with comparisons between males and females reported for each genotype. The selected models (lowest AIC) are highlighted in bold.

| **a) Genotype level** | | | |  |  |  |
| --- | --- | --- | --- | --- | --- | --- |
| Model | **df** | **AIC** | |  |  |  |
| Beta distribution | **4** | -**10799.88** | |  |  |  |
| Gamma distribution | 4 | -10.230.66 | |  |  |  |
|  |  |  | |  |  |  |
| Contrast | **Estimate** | | **SE** | | **z** | ***P*** |
| WW-Wy | 0.111 | | 0.008 | | 13.084 | **<0.001** |
| Wy-yy | -0.014 | | 0.003 | | -5.220 | **<0.001** |
| WW-yy | 0.096 | | 0.008 | | 11.486 | **<0.001** |

| **b) Sex level** | | |
| --- | --- | --- |
| **Model** | **df** | **AIC** |
| **WW genotype** | | |
| Beta distribution | **3** | **-500.264** |
| Gamma distribution | 3 | -459.729 |
| males vs females; Estimate = 0.113 ± 0.099, z = 1.14, *P* = 0.254 | | |
|  | | |
| **Wy genotype** | | |
| Beta distribution | **3** | **-4204.602** |
| Gamma distribution | 3 | -4068.919 |
| males vs females; Estimate = -0.183 ± 0.069, z = -2.623, *P* = 0.009 | | |
|  | | |
| **yy genotype** | | |
| Beta distribution | **3** | **-6202.65** |
| Gamma distribution | 3 | -5924.53 |
| males vs females; Estimate = 0.415 ± 0.051, z = 8.187, *P* = 2.68e-16 | | |

**Table Supp. Info 2.** Sample size of the individuals tested and all possible pair combinations used in the experiments. In addition, the table below reports the crossing scheme for the precopulatory stage mating experiment (left genotype = male; right genotype = female)

|  | **Treatment** | **Mating success** | **Oviposition & Hatching day** | **Unsuccessful mating** |
| --- | --- | --- | --- | --- |
| **Males** | **WW** | 87 | 39 | - |
|  | **Wy** | 61 | 415 | - |
|  | **yy** | 112 | 557 | - |
| **Females** | **WW** | 73 | 44 | - |
|  | **Wy** | 53 | 366 | - |
|  | **yy** | 89 | 495 | - |
| **No. of *yellow* allele** | **0 (WWxWW)** | 41 | 1 | - |
|  | **1 (WWxWy)** | 34 | 12 | - |
|  | **2 (WWxyy OR WyxWy)** | 56 | 164 | - |
|  | **3 (Wyxyy)** | 29 | 355 | - |
|  | **4 (yyxyy)** | 47 | 225 | - |
| **Pair type (W- individuals are either WW or Wy)** | **♀ W-allele + ♂ yy** | 38 | 266 | 107 |
|  | **♀ W-allele + ♂ W-allele** | 121 | 166 | 210 |
|  | **♀ yy + ♂ W-allele** | 44 | 214 | 114 |
|  | **♀ yy + ♂ yy** | 47 | 225 | 130 |

| **Genotype combination** | **N** |
| --- | --- |
| **WW x WW** | 41 |
| **Wy x Wy** | 19 |
| **yy x yy** | 47 |
| **WW x Wy** | 17 |
| **WW x yy** | 22 |
| **Wy x WW** | 17 |
| **Wy x yy** | 15 |
| **yy x WW** | 15 |
| **yy x Wy** | 14 |

*** Total number of pairs with fully known genotype is 207; the remaining 85 pairs included individuals with incomplete genotype**

**Table Supp. Info 3.** The table below reports the model selection for the analyzes that tested whether the heterozygote advantage was linked to the sire or dam’s side. The selected model (lowest AIC) is highlighted in bold.

| **Fecundity** | | |
| --- | --- | --- |
| Model | **df** | **AIC** |
| Poisson distribution | 2 | 4981.001 |
| **Negative binomial distribution** | **3** | **1211.366** |
|  | | |
| **Fertility** | | |
| Model | **df** | **AIC** |
| Poisson distribution | 2 | 6499.053 |
| **Negative binomial distribution** | **3** | **1273.759** |

**Table Supp. Info 4** The upper table below reports the model selection for the oviposition and hatching day considering both models at the individual level (males and females) and at the pair level (number of yellow alleles in the pair and the pair type). The selected model (lowest AIC) is highlighted in bold. The lower table reports the results for the oviposition day and hatching day analyzes.

| **Model selection** | | | | | | | | |  |
| --- | --- | --- | --- | --- | --- | --- | --- | --- | --- |
|  | **Oviposition day** | | | | **Hatching day** | | | |  |
|  | **Poisson distribution** | | **Negative binomial distribution** | | **Poisson distribution** | | **Negative binomial distribution** | |  |
|  | Df | AIC | Df | AIC | Df | AIC | Df | AIC | |
| Males | **4** | **2548.382** | 5 | 2550.386 | **4** | **4062.084** | 5 | 4064.088 | |
| Females | **4** | **2304.378** | 5 | 2306.382 | **4** | **3688.296** | 5 | 3690.300 | |
| N of y allele | **6** | **2109.264** | 7 | 2111.267 | **6** | **3406.006** | 7 | 3408.009 | |
| Pair type | **5** | **2437.764** | 6 | 2439.769 | **5** | **3868.586** | 6 | 3870.590 | |

| **Chi-square test** | | | | | | | | |
| --- | --- | --- | --- | --- | --- | --- | --- | --- |
|  | **Oviposition day** | | | | **Hatching day** | | | |
|  | **Chisq** | **Df** | ***P*** | **Random effect (0.37%)** | **Chisq** | **Df** | ***P*** | **Random effect (0.07%)** |
| Male genotype | 1.6326 | 2 | 0.4421 | 0.01, 0.11 | 0.2197 | 2 | 0.896 | 0.002, 0.048 |
| Female genotype | 3.8032 | 2 | 0.1493 | 0.01, 0.11 | 0.7597 | 2 | 0.684 | 0.002, 0.049 |
| N yellow alleles | 3.8134 | 4 | 0.4319 | 0.01, 0.11 | 0.6222 | 4 | 0.9606 | 0.002, 0.047 |
| Pair type | 4.6074 | 3 | 0.2029 | 0.01, 0.11 | 1.1349 | 3 | 0.7687 | 0.002, 0.049 |

**Table Supp. Info 5** Table a) reports the effect of the genotype, weight and age on the mating probability, for males (upper part) and females (lower part) separately through Chi-square test. Table b) reports the main effects of genotype, generation, age and weight on the mating delay of males (top table) and females (bottom table) through LR-test.

| **a) Males** | | | | | |
| --- | --- | --- | --- | --- | --- |
|  | **Df** | **Deviance** | **Residual Df** | **Residual Deviance** | ***P*** |
| Male genotype | 2 | 1.9281 | 254 | 350.10 | 0.3814 |
| Male weight | 1 | 1.3695 | 253 | 348.73 | 0.2419 |
| Male age | 1 | 3.3057 | 252 | 345.43 | 0.0690 |

| **Females** | | | | | |
| --- | --- | --- | --- | --- | --- |
|  | **Df** | **Deviance** | **Residual Df** | **Residual Deviance** | ***P*** |
| Female genotype | 2 | 1.3533 | 209 | 286.40 | 0.5083 |
| Female weight | 1 | 10.6629 | 208 | 275.74 | **0.0011** |
| Female age | 1 | 0.1517 | 207 | 275.58 | 0.6969 |

| **b) Males** | | | |
| --- | --- | --- | --- |
|  | **LR Chi-square** | **Df** | ***P*** |
| Male genotype | 2.8395 | 2 | 0.2418 |
| Generation | 14.5574 | 2 | **0.0007** |
| Male age | 0.0658 | 1 | 0.7975 |
| Male weight | 0.7538 | 1 | 0.3853 |

| **Females** | | | |
| --- | --- | --- | --- |
|  | **LR Chi-square** | **Df** | ***P*** |
| Female genotype | 3.4934 | 2 | 0.1744 |
| Generation | 12.2033 | 2 | **0.0022** |
| Female age | 2.6574 | 1 | 0.1031 |
| Female weight | 1.1457 | 1 | 0.2845 |

**Table Supp. Info 6.** The upper part of the table reports the effect of the number of *yellow* allele and the pair type on the copulation probability through LR-test. The lower part of the table reports the output of the GLM for the number of *yellow* allele in the pair, with the ‘0 yellow allele’ pair as reference (i.e. the intercept).

| **Chi-square** | | | | | |
| --- | --- | --- | --- | --- | --- |
|  | **Df** | **Deviance** | **Residual Df** | **Residual Deviance** | ***P*** |
| No. yellow allele in pair | 4 | 12.996 | 202 | 269.31 | **0.0113** |
| Pair type | 3 | 3.6337 | 243 | 329.78 | 0.3038 |

| **GLM Output** | | | | |
| --- | --- | --- | --- | --- |
|  | **Estimate** | **Std. Error** | **z** | ***P*** |
| **Intercept** | 1.2685 | 0.3773 | 3.362 | **0.0008** |
| 1 yellow allele | -1.6252 | 0.5136 | -3.164 | **0.0016** |
| 2 yellow alleles | -1.2685 | 0.4624 | -2.743 | **0.0061** |
| 3 yellow alleles | -1.0609 | 0.5308 | -1.999 | **0.0457** |
| 4 yellow alleles | -0.7916 | 0.4821 | -1.642 | 0.1006 |

**Table Supp. Info 7.** The upper table reports the effect of the number of *yellow* allele and the pair type on the mating delay through LR-test. Although no significant effect of the pair type was found, we report the output of the Cox model because a closer look at the model output showed a significant effect. The intercept of this last analysis is set on the pair type “female yy + male W-allele”.

| **LR-test** | | | |
| --- | --- | --- | --- |
|  | **LR Chi-square** | **Df** | ***P*** |
| No. *yellow* allele in pair | 2.2647 | 4 | 0.6872 |
| Pair type | 7.0846 | 3 | 0.0693 |

| **Cox model** | | | | | |
| --- | --- | --- | --- | --- | --- |
|  | **Coeff** | **Exp(coeff)** | **se(coeff)** | **z** | ***P*** |
| female W-allele + male W-allele | -0.6006 | 0.5485 | 0.2519 | -2.384 | **0.0171** |
| female W-allele + male yy | -0.7378 | 0.4782 | 0.3151 | -2.341 | **0.0192** |
| female yy + male yy | -0.7554 | 0.4698 | 0.2996 | -2.522 | **0.0117** |

**Table Supp. Info 8.** Table a) reports the model selection for the fecundity, fertility and hatching success traits for males and females. The selected model (lowest AIC) is highlighted in bold. Tables b), c), d) report the type III analyzes of variance through Chi-square of the fecundity (b), fertility (c) and hatching success (d) GLMMs showing the effect of the interactions ‘genotype * weight’ and ‘genotype * weight’.

**a) Fecundity, fertility and hatching success model selection**

|  | **Model** | **df** | **Fecundity AIC** | **Fertility AIC** | **Hatching success AIC** |
| --- | --- | --- | --- | --- | --- |
| **Males** | glmer.poisson | 10 | 58577.68 | 85293.79 | 36947.74 |
|  | glmer.nb | 11 | 13426.97 | 12225.77 | 9743.11 |
|  | zeroinfl.poisson | 15 | 40502.33 | 27806.33 | 11069.94 |
|  | **zeroinfl.nb** | **16** | **12696.22** | **11070.99** | **8412.43** |
|  | | | | |  |
| **Females** | glm.poisson | 10 | 35030.23 | 58006.22 | 25897.53 |
|  | glm.nb | 11 | 10354.22 | 10285.23 | 8091.68 |
|  | zeroinfl.poisson | 15 | 25587.29 | 23518.50 | 10351.16 |
|  | **zeroinfl.nb** | **16** | **9851.07** | **9348.08** | **7106.43** |

**b) Fecundity**

| **Males** | | | |
| --- | --- | --- | --- |
|  | **Df** | **Chisq** | ***P*** |
| Intercept | 1 | 2323.33 | **<2.2e-16** |
| Genotype | 2 | 8.725 | **0.013** |
| Weight | 1 | 0.631 | 0.427 |
| Inbreeding Coefficient | 1 | 10.496 | **0.001** |
| Genotype : Weight | 2 | 1.247 | 0.536 |
| Genotype : Inbreeding Coefficient | 2 | 3.658 | 0.161 |

| **Females** | | | |
| --- | --- | --- | --- |
|  | **Df** | **Chisq** | ***P*** |
| Intercept | 1 | 3960.02 | **<2.2e-16** |
| Genotype | 2 | 10.49 | **0.005** |
| Weight | 1 | 7.60 | **0.006** |
| Inbreeding Coefficient | 1 | 4.081 | **0.043** |
| Genotype : Weight | 2 | 0.540 | 0.763 |
| Genotype : Inbreeding Coefficient | 2 | 4.123 | 0.127 |

**c) Fertility**

| **Males** | | | |
| --- | --- | --- | --- |
|  | **Df** | **Chisq** | ***P*** |
| Intercept | 1 | 26719.99 | **<2-16** |
| Genotype | 2 | 0.479 | 0.787 |
| Weight | 1 | 1.075 | 0.299 |
| Inbreeding Coefficient | 1 | 0.784 | 0.376 |
| Genotype : Weight | 2 | 1.445 | 0.486 |
| Genotype : Inbreeding Coefficient | 2 | 3.021 | 0.221 |

| **Females** | | | |
| --- | --- | --- | --- |
|  | **Df** | **Chisq** | ***P*** |
| Intercept | 1 | 14689.49 | **<2.2e-16** |
| Genotype | 2 | 1.579 | 0.454 |
| Weight | 1 | 27.840 | **1.318e-07** |
| Inbreeding Coefficient | 1 | 0.559 | 0.455 |
| Genotype : Weight | 2 | 0.012 | 0.994 |
| Genotype : Inbreeding Coefficient | 2 | 2.051 | 0.359 |

**d) Hatching success**

| **Males** | | | |
| --- | --- | --- | --- |
|  | **Df** | **Chisq** | ***P*** |
| Intercept | 1 | 31757.26 | **<2e-16** |
| Genotype | 2 | 0.302 | 0.859 |
| Weight | 1 | 1.75 | 0.186 |
| Inbreeding Coefficient | 1 | 0.984 | 0.321 |
| Genotype : Weight | 2 | 0.415 | 0.813 |
| Genotype : Inbreeding Coefficient | 2 | 5.247 | 0.073 |

| **Females** | | | |
| --- | --- | --- | --- |
|  | **Df** | **Chisq** | ***P*** |
| Intercept | 1 | 15369.44 | **<2e-16** |
| Genotype | 2 | 2.449 | 0.294 |
| Weight | 1 | 0.346 | 0.556 |
| Inbreeding Coefficient | 1 | 0.928 | 0.336 |
| Genotype : Weight | 2 | 0.664 | 0.718 |
| Genotype : Inbreeding Coefficient | 2 | 1.391 | 0.499 |

**Table Supp. Info 9.** Table a) reports the effect of genotype, weight and inbreeding coefficient on the fecundity of males and females. Tables b) and d) report genotype pairwise comparisons for, respectively, males and females and Tables c) and e) report the GLMM models output for males and females’ fecundity.

| **a) Males** | | | |
| --- | --- | --- | --- |
|  | **Df** | **Chisq** | ***P*** |
| Genotype | 2 | 6.462 | **0.039** |
| Weight | 1 | 0.154 | 0.695 |
| Inbreeding Coefficient | 1 | 37.886 | **7.501e-10** |

| **Females** | | | |
| --- | --- | --- | --- |
|  | **Df** | **Chisq** | ***P*** |
| Genotype | 2 | 9.045 | **0.011** |
| Weight | 1 | 90.359 | **<2.2e-16** |
| Inbreeding Coefficient | 1 | 8.781 | **0.003** |

**MALES:**

**b) Pairwise comparisons based on estimated marginal means; Tukey-HSD adjustment**

| **Contrast** | **Estimate** | **SE** | **df** | **t** | ***P*** |
| --- | --- | --- | --- | --- | --- |
| WW-Wy | 0.048 | 0.076 | 1089 | 0.627 | 0.805 |
| yy-Wy | -0.089 | 0.043 | 1089 | -2.047 | 0.102 |
| yy-WW | -0.136 | 0.072 | 1089 | -1.893 | 0.141 |

| **c) Zero inflated**; Intercept = yy genotype | | | | | |
| --- | --- | --- | --- | --- | --- |
|  | **Estimate** | **Std. Error** | **z** | ***P*** |  |
| Count model | | | | |  |
| Intercept | 5.158 | 0.026 | 199.33 | **<2e-16** |  |
| WW genotype | 0.136 | 0.072 | 1.89 | 0.058 |  |
| Wy genotype | 0.088 | 0.043 | 2.05 | **0.041** |  |
| Weight | 0.009 | 0.022 | 0.39 | 0.695 |  |
| Inbreeding coefficient | -0.132 | 0.022 | -6.16 | **7.5e-10** |  |
|  | | | | | |
| Zero inflated model | | | | | |
| Intercept | -2.066 | 0.124 | -16.66 | **<2e-16** |  |
| WW genotype | -1.373 | 0.618 | -2.222 | **0.026** |  |
| Wy genotype | -0.768 | 0.267 | -2.883 | **0.004** |  |
| Weight | -0.096 | 0.117 | -0.819 | 0.413 |  |
| Inbreeding coefficient | -0.056 | 0.114 | -0.493 | 0.662 |  |

**FEMALES:**

| **d) Contrast** | **Estimate** | **SE** | **df** | **t** | ***P*** |
| --- | --- | --- | --- | --- | --- |
| Wy-WW | 0.098 | 0.064 | 835 | 1.526 | 0.279 |
| Wy-yy | 0.132 | 0.044 | 835 | 3.003 | **0.008** |
| WW-yy | 0.033 | 0.058 | 835 | 0.568 | 0.837 |

| **e) Zero inflated**; Intercept = Wy genotype | | | | | |
| --- | --- | --- | --- | --- | --- |
|  | **Estimate** | **Std. Error** | **z** | ***P*** |  |
| Count model | | | | | |
| Intercept | 5.294 | 0.035 | 150.65 | **<2e-16** |  |
| WW genotype | -0.098 | 0.064 | -1.53 | 0.126 |  |
| yy genotype | -0.131 | 0.044 | -3.00 | **0.003** |  |
| Weight | 0.200 | 0.021 | 9.51 | **<2e-16** |  |
| Inbreeding coefficient | -0.064 | 0.022 | -2.96 | **0.003** |  |
|  | | | | | |
| Zero inflated model | | | | | |
|  |  |  |  |  |  |
| Intercept | -4.093 | 0.505 | -8.094 | **5.8e-16** |  |
| WW genotype | 0.656 | 0.699 | 0.938 | 0.348 |  |
| yy genotype | 1.446 | 0.539 | 2.683 | **0.007** |  |
| Weight | 0.265 | 0.169 | 1.571 | 0.116 |  |
| Inbreeding coefficient | 0.267 | 0.177 | 1.514 | 0.129 |  |

**Table Supp. Info 10.** Table a) reports the effect of genotype, weight and inbreeding coefficient on the fertility of males and females. Table b) reports genotype pairwise comparisons and Table c) reports the GLMM models output for males’ fertility.

| **a) Males** | | | |
| --- | --- | --- | --- |
|  | **Df** | **Chisq** | ***P*** |
| Genotype | 2 | 1.229 | 0.541 |
| Weight | 1 | 0.788 | 0.375 |
| Inbreeding Coefficient | 1 | 5.273 | **0.022** |

| **Females** | | | |
| --- | --- | --- | --- |
|  | **Df** | **Chisq** | ***P*** |
| Genotype | 2 | 0.922 | 0.631 |
| Weight | 1 | 71.761 | **<2e-16** |
| Inbreeding Coefficient | 1 | 0.150 | 0.699 |

**MALES:**

**b) Pairwise comparisons based on estimated marginal means; Tukey-HSD adjustment**

| **Contrast** | **Estimate** | **SE** | **df** | **t** | ***P*** |
| --- | --- | --- | --- | --- | --- |
| yy-WW | 0.095 | 0.087 | 1188 | 1.092 | 0.519 |
| yy-Wy | 0.021 | 0.049 | 1188 | 0.423 | 0.906 |
| WW-Wy | -0.074 | 0.089 | 1188 | -0.828 | 0.686 |

| **c) Zero inflated**; Intercept = yy genotype | | | | | |
| --- | --- | --- | --- | --- | --- |
|  | **Estimate** | **Std. Error** | **z** | ***P*** |  |
| Count model | | | | | |
| Intercept | 5.085 | 0.031 | 163.57 | **<2e-16** |  |
| WW genotype | -0.095 | 0.087 | -1.09 | 0.275 |  |
| Wy genotype | -0.021 | 0.049 | -0.42 | 0.672 |  |
| Weight | 0.022 | 0.025 | 0.89 | 0.375 |  |
| Inbreeding coefficient | -0.060 | 0.026 | -2.30 | **0.022** |  |
|  | | | | | |
| Zero inflated model | | | | | |
| Intercept | -0.479 | 0.078 | -6.117 | **9.51e-10** |  |
| WW genotype | -0.774 | 0.245 | -3.153 | **0.002** |  |
| Wy genotype | -0.832 | 0.148 | -5.639 | **1.71e-08** |  |
| Weight | 0.136 | 0.069 | 1.960 | 0.05 |  |
| Inbreeding coefficient | 0.332 | 0.070 | 4.775 | **1.80e-06** |  |

**Table Supp. Info 11.** Table a) reports the effect of genotype, weight and inbreeding coefficient on the hatching success of males and females. Tables b) and d) report genotype pairwise comparisons for, respectively, males and females and Tables c) reports the GLMM output for males and females’ hatching success.

| **a) Males** | | | |
| --- | --- | --- | --- |
|  | **Df** | **Chisq** | ***P*** |
| Genotype | 2 | 2.432 | 0.296 |
| Weight | 1 | 5.594 | **0.018** |
| Inbreeding Coefficient | 1 | 0.003 | 0.954 |

| **Females** | | | |
| --- | --- | --- | --- |
|  | **Df** | **Chisq** | ***P*** |
| Genotype | 2 | 1.939 | 0.379 |
| Weight | 1 | 0.0003 | 0.986 |
| Inbreeding Coefficient | 1 | 0.054 | 0.817 |

**MALES:**

**b) Pairwise comparisons based on estimated marginal means, Tukey-HSD adjustment**

| **Contrast** | **Estimate** | **SE** | **df** | **t** | ***P*** |
| --- | --- | --- | --- | --- | --- |
| yy-WW | 0.100 | 0.065 | 1092 | 1.552 | 0.267 |
| yy-Wy | 0.008 | 0.038 | 1092 | 0.216 | 0.975 |
| WW-Wy | -0.092 | 0.067 | 1092 | -1.379 | 0.352 |

| **c) Zero inflated**; Intercept = yy genotype | | | | | |
| --- | --- | --- | --- | --- | --- |
|  | **Estimate** | **Std. Error** | **z** | ***P*** |  |
| Count model | | | | | |
| Intercept | 4.281 | 0.024 | 177.77 | **<2e-16** |  |
| WW genotype | -0.100 | 0.065 | -1.55 | 0.121 |  |
| Wy genotype | -0.008 | 0.038 | -0.22 | 0.829 |  |
| Weight | -0.047 | 0.020 | -2.37 | **0.018** |  |
| Inbreeding coefficient | 0.001 | 0.020 | 0.06 | 0.954 |  |
|  | | | | | |
| Zero inflated model | | | | | |
| Intercept | -0.359 | 0.080 | -4.481 | **7.41e-06** |  |
| WW genotype | -0.821 | 0.246 | -3.330 | **0.0009** |  |
| Wy genotype | -0.810 | 0.150 | -5.387 | **7.16e-08** |  |
| Weight | 0.161 | 0.071 | 2.283 | **0.022** |  |
| Inbreeding coefficient | 0.280 | 0.070 | 3.983 | **6.81e-05** |  |

**Table Supp. Info 12.** The table reports the pairwise comparisons between pair type for the mating success.

**Pairwise comparisons based on estimated marginal means; Tukey HSD adjustment**

| **Contrast** | **Estimate** | **SE** | **z** | ***P*** |
| --- | --- | --- | --- | --- |
| **♀**W-allele + ♂yy *vs* ♀W-allele + ♂W-allele | 0.1926 | 0.0327 | 5.888 | **<0.0001** |
| ♀W-allele + ♂yy *vs* ♀yy + ♂W-allele | 0.4139 | 0.0437 | 9.477 | **<0.0001** |
| ♀W-allele + ♂yy *vs* ♀yy + ♂yy | 0.4667 | 0.0402 | 11.604 | **<0.0001** |
| ♀W-allele + ♂W-allele *vs* ♀yy + ♂W-allele | 0.2213 | 0.0369 | 5.996 | **<0.0001** |
| ♀W-allele + ♂W-allele *vs* ♀yy + ♂yy | 0.2741 | 0.0335 | 8.182 | **<0.0001** |
| ♀yy + ♂W-allele *vs* ♀yy + ♂yy | 0.0527 | 0.0426 | 1.239 | 0.6022 |

References

Cacoyianni, Z., Kovacs, I. V., & Hoffmann, A. A. (1995). Laboratory Adaptation and Inbreeding in Helicoverpa-Punctigera (Lepidoptera, Noctuidae). *Australian journal of zoology*, *43*(1), 83-90.

Roush, R. T. (1986). Inbreeding depression and laboratory adaptation in Heliothis virescens (Lepidoptera: Noctuidae). *Annals of the Entomological Society of America*, *79*(4), 583-587.
